# Use of activated enol ethers in the synthesis of pyrazoles: reactions with hydrazine and a study of pyrazole tautomerism

**DOI:** 10.3762/bjoc.10.70

**Published:** 2014-04-01

**Authors:** Denisa Tarabová, Stanislava Šoralová, Martin Breza, Marek Fronc, Wolfgang Holzer, Viktor Milata

**Affiliations:** 1 Department of Organic Chemistry, Faculty of Chemical and Food Technology, Slovak University of Technology, Radlinského 9, SK-812 37 Bratislava, Slovakia; 2 Department of Physical Chemistry, Faculty of Chemical and Food Technology, Slovak University of Technology, Radlinského 9, SK-812 37 Bratislava, Slovakia; 3 Department of Pharmaceutical Chemistry, Faculty of Pharmacy, Comenius University in Bratislava, Odbojárov 10, SK-832 32 Bratislava, Slovakia; 4 Department of Drug and Natural Product Synthesis, Vienna University, Althanstrasse 14, A-1090 Vienna, Austria

**Keywords:** bis-enehydrazines, enol ethers, NMR, pyrazole, tautomerism

## Abstract

Activated enol ethers derived from esters or the dinitrile of malonic acid, or from pentane-2,4-dione were treated with hydrazine hydrate. The structures of the obtained products – pyrazoles **5** – were studied with a focus on tautomerism and supramolecular structure. A reverse addition of the reagents led to the isolation of two novel products, namely bis-enehydrazines **6** with an unsymmetrical arrangement of the formally equivalent subunits.

## Introduction

Enol ethers are reactive species frequently used in organic synthesis [1]. They react predominantly with electrophiles. The introduction of one or more strong electron-withdrawing groups at the other end of the double bond from the alkoxy group causes an inversion of electron demand. These activated enol ethers react surprisingly easily with various nucleophiles such as amines, thiols, alcohols or C-anions [2] under the conditions of nucleophilic vinylic substitution [3]. When using bi- or trifunctional nucleophiles, cyclic or bicyclic products are formed. Activated enol ethers thus represent trifunctional electrophiles and are useful building blocks for the introduction of a three carbon fragment into the resulting molecule. Thus, the reaction of enol ethers with hydrazines produces pyrazoles, with amidines pyrimidines, with hydroxylamine isoxazoles, and with anilines quinolines/ones are formed [2].

**Scheme 1:**
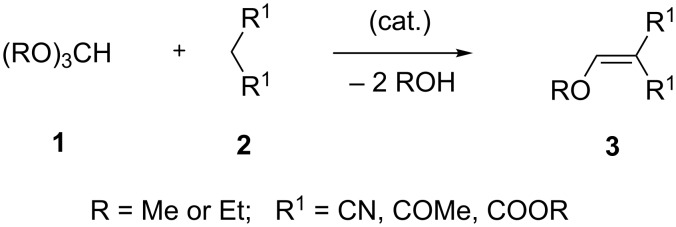
Preparation of enol ethers.

Electron-accepting groups can include ester, cyano, acetyl, nitro and trifluoroacetyl moieties. Thus, the simplest way to obtain activated enol ethers **3** is the condensation of active methylene components **2** such as malonic acid derivatives (esters, nitrile), (trifluoro)acetoacetic acid derivatives (esters, nitrile), acetylacetone, and nitroacetates with trialkyl orthoformate **1** (Scheme 1). Transesterification can be avoided if **1** bears the same alkyl group as **2**. The reaction can be carried out with or without Lewis acid catalysis [2].

Pyrazoles constitute a large group of pharmacologically active compounds and are mostly prepared by reacting hydrazines with suitable 1,3-dielectrophiles. The synthesis of pyrazoles and related compounds can be implemented by four different approaches:

1. reaction of (substituted) hydrazines with β-functional compounds or their equivalents (the most frequently used method),
2. 1,3-dipolar cycloaddition with diazo compounds as a source of N–N linkage,
3. degradation of fused pyrazoles or
4. rearrangement of other monocyclic heterocycles under chemical, thermal or photochemical conditions [4].

**Scheme 2:**
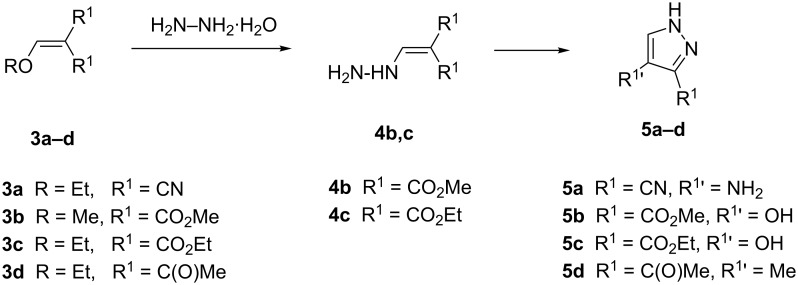
Reaction of enol ethers with hydrazine hydrate.

Upon reaction of hydrazine with symmetrical enol ethers, only a single product is formed, which is capable of annular prototropic tautomerism [5–6]. In contrast, the use of „asymmetric“ enol ethers, that is, R^1^, R^1^ in **2** and **3** are not equal, may lead to two different pyrazoles, both of which exhibit tautomerism). However, depending on the different reactivities of the electron-withdrawing groups R^1^ only a single product is formed (Scheme 2) [7].

## Results and Discussion

In this work we have studied reactions of symmetric enol ethers with hydrazine hydrate according to Scheme 2. The enol ethers ethoxymethylidenemalononitrile (EMMN, **3a**), dimethyl methoxymethylidenemalonate (MMMM, **3b**)**,** diethyl ethoxymethylidenemalonate (EMME, **3c**), and 3-(ethoxymethylidene)pentane-2,4-dione (EMAA, **3d**) were employed. Three-component reactions (hydrazine + trialkylorthoformate + active methylidene component) were not studied.

**Scheme 3:**
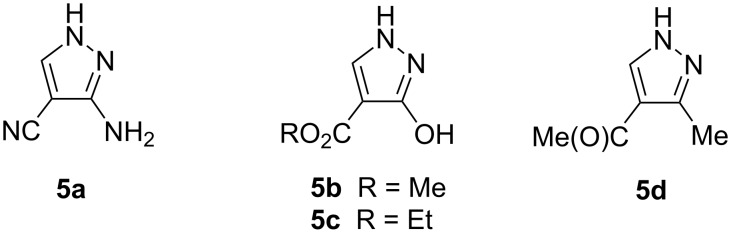
Pyrazoles **5**.

The products resulting from the reaction of a hydrazine hydrate with activated symmetrical enol ethers **3** are shown in Scheme 3.

**Figure 1:**
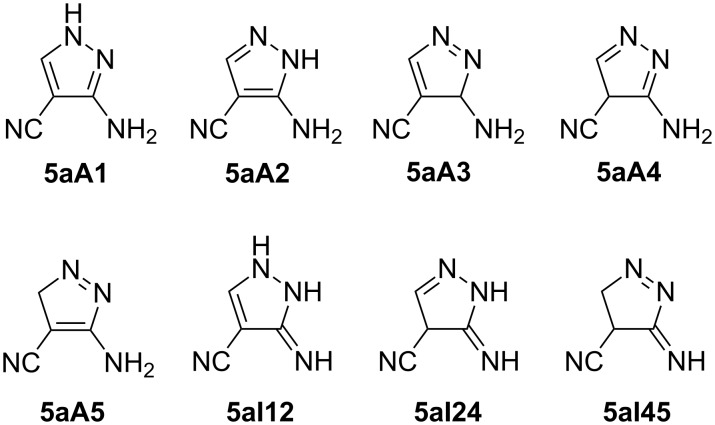
Tautomers of **5a**.

It is well known that the reaction of hydrazine hydrate with alkoxymethylidenemalononitrile affords 3-amino-1*H*-pyrazole-4-carbonitrile (**5a**) [8–13]. Eight tautomeric forms of **5a** could theoretically be expected (Figure 1). Five of these tauromeric forms carry an amino substituent, which is denoted by the third character being an A in the numbering of these tautomers and the fourth number giving the position of one hydrogen. Three of the tautomeric forms of **5a** are characterized by an exocyclic imino function. This is denoted by the third character I in the numbering and the fourth and fifth numbers giving the positions of two hydrogens.

**Figure 2:**
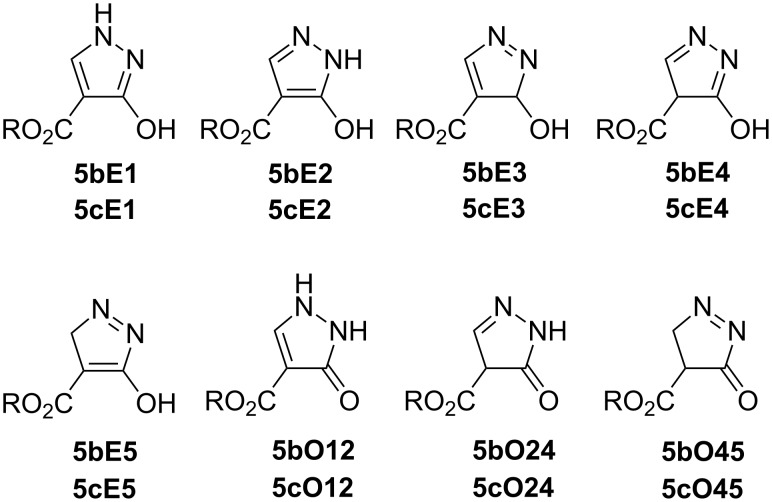
Tautomers of **5b** (R = Me), **5c** (R = Et).

When dimethyl methoxymethylidenemalonate (**3b**) is used, methyl 3-hydroxy-1*H*-pyrazole-4-carboxylate (**5b**) is formed. Analogously, reaction of diethyl ethoxymethylenemalonate (**3c**) gives the corresponding ethyl 3-hydroxy-1*H*-pyrazole-4-carboxylate (**5c**) [14–18]. The Reaction of enol ethers **3b** and **3c** with hydrazine hydrate at rt for 10 min gave products of an S_N_V reaction, **4b** and **4c**. These then underwent intramolecular cyclization to yield **5b** and **5c**. Due to oxo–enol tautomerism eight tautomers are theoretically possible (Figure 2). The labeling scheme of the tautomers of **5b** uses an E to indicate the (en)ol form and an O to indicate the oxo form.

**Figure 3:**
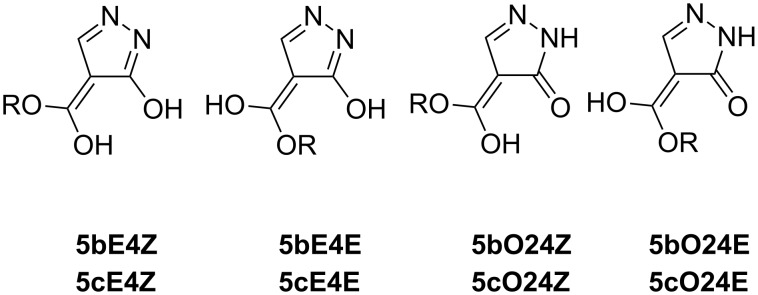
Tautomers of **5b** (R = Me), **5c** (R = Et).

However, more tautomeric forms of the ester species are theoretically possible as the C-4 substituent can also be involved in tautomerism. For instance, additional species, which are possible for ester compounds **5b** and **5c** are shown in Figure 3, with the last character of the label being an E indicates enol, O denotes oxo, and (*Z*,*E*) – HO vs OH/=O

**Figure 4:**
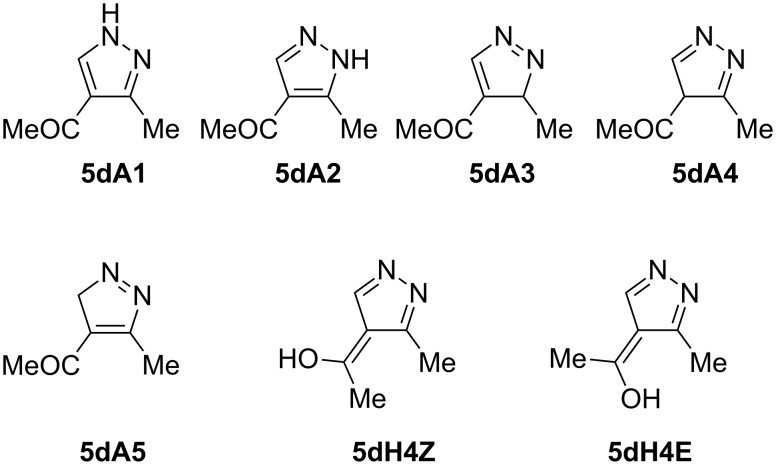
Tautomers of **5d**.

Compound **5b** was previously prepared by a ring contraction of pyrimidine-2,4-dione [19]. It is known from the literature that after the reaction of ethoxymethylideneacetylacetone (**3d**) with hydrazine hydrate 1-(3-methyl-1*H*-pyrazol-4-yl)ethanone (**5d**) is produced [20]. The latter bears the tautomerizable acetyl group so that the tautomeric characteristics are different from the previous case and similar to that of **5a** (Figure 4). Here, Z/*E* refers to the mutual positions of methyl groups.

Compared to the procedure described by Mitkidou et al. [20], we were able to prepare **5d** in one step, in a shorter time (1 h heated under reflux in EtOH) and with a higher yield of 88%. Mitkidou et al. prepared **5d** in a two-step synthesis [20] (8 h heated under reflux in toluene 55% yield or 2 h in CHCl_3_) in 69% yield. Other preparations of **5d** were carried out in aqueous media [21–23].

Enol ethers with different alkoxy groups afforded the same products. Reactions were carried out in ethanol or without a solvent. If the ratio enol ether/hydrazine is 1:1, as in the case of the reaction with **3c**, the formation of 7-aminopyrazolo[1,5-*a*]pyrimidine-3,6-dicarbonitrile could be expected as a byproduct [24–25]. When hydrazine hydrochloride is used [14], ethyl 3-ethoxypyrazole-4-carboxylate was obtained in 41% yield and the expected ethyl 3-oxo-2,3-dihydropyrazole-4-carboxylate (**5c**, oxo form) in 37% yield. The first product was obtained as an oil, whereas compound **5c** was afforded as a white powder.

**Scheme 4:**
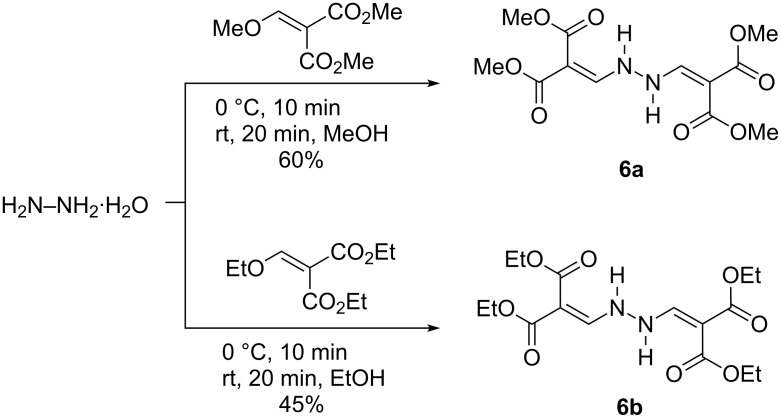
Reactions of hydrazine hydrate with dialkyl alkoxymethylidenemalonates.

**Figure 5:**
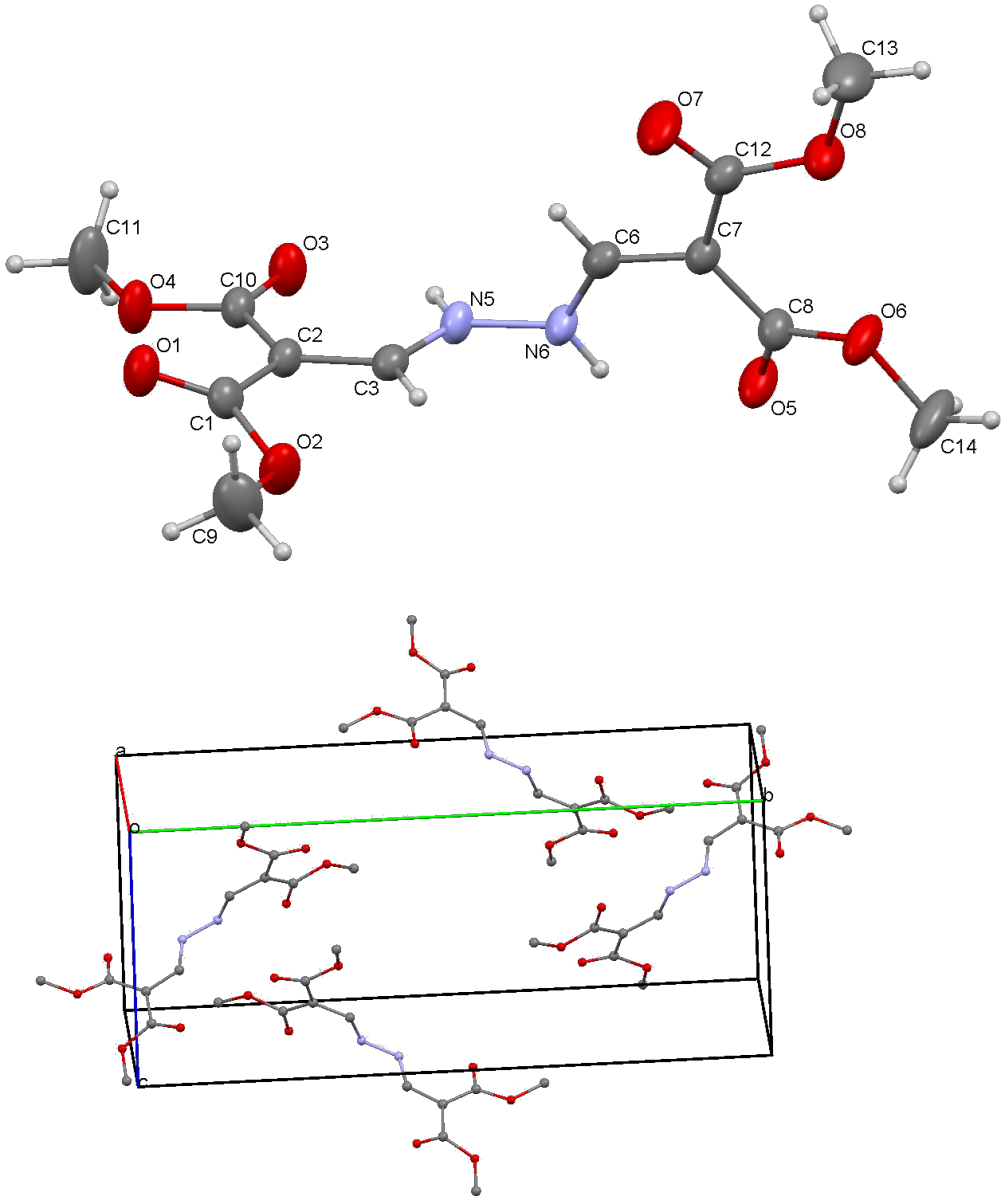
Crystal structure and crystal packing of compound **6a**.

The reversal of the addition by adding hydrazine hydrate dropwise to the stirred enol ether led to the bis-*N*,*N*´-product **6a** in 60% yield as a colorless crystalline product and **6b** (43% yield) as yellowish crystals. This product could not be thermally cyclized into the corresponding pyrazole derivative, presumably due to the reduced nucleophilicity of the hydrazine nitrogen bearing the electron-deficient vinyl substituent (Scheme 4). The structure of **6a** was confirmed by spectroscopic analysis, elemental analysis, and X-ray structure analysis (Figure 5).

A sample of compound **6a** was recrystallized by slow evaporation from chloroform. X-ray diffraction data (Supporting Information File 1, Tables S1–S5) were collected on an Oxford Diffraction Gemini R diffractometer equipped with a Ruby CCD detector and Mo Kα sealed-tube source at rt. Data reduction was performed with Oxford Diffraction *CrysAlis RED version 1.171.33.42* software [26]. The crystal structure was solved and refined with SHELXS97 [27].

The molecule consists of the two parts rotated around the N–N bond, with the torsion angle C3–N5–N6–C6 −77.0(2)°, while the N–N bond and adjacent C=C bonds are highly planar with torsion angles 174.09(15)° for C2–C3–N5–N6 and 177.71(16)° for N5–N6–C6–C7. Correspondent bond lengths in the molecule show only minor variations. Three carbonyl bonds in the ester groups display a *Z* conformation with regard to the carbon atom of the double bond (O5–C8–O6–C14 and O7–C12–O8–C13 to C6 and O3–C10–O4–C11 to C3), while the fourth carbonyl group exhibits an *E* conformation (O1–C1–O2–C9 to C3). Torsion angles between the carbonyl and double C=C bond are 15.9(3)°, 11.1(3)°, 0.6(3)° for the first three ester groups (C6–C7–C8–O5, C6–C7–C12–O7, C3–C2–C10–O3) and 152.28(17)° for the last one (O1–C1–C2–C3) (Supporting Information File 1, Table S5).

N5–H5A forms an intramolecular hydrogen bond with O3 and an intermolecular hydrogen bond with O4. N6–H6A forms an intramolecular hydrogen bond with O5, while both also form an intermolecular hydrogen bond with O5 from the next molecule as shown in Table S2. These two hydrogen bonds are in a rhomboid arrangement with angles H6A–O5–H6A 86.10(5)° and O5–H6A–O5 93.90(5)°. Selected interatomic distances, bond and torsion angles are presented in Supporting Information File 1, Tables S2, S4 and S5.

### NMR measurements

The spectra of compounds **5a–d** are characterized by broad to very broad resonance lines, hinting at dynamic processes related to prototropic tautomerism. It was attempted to record spectra in CDCl_3_ – where a rapid exchange between tautomeric forms was expected – and in DMSO-*d*_6_ solution – where we assumed slower interconversion rates due to the acceptor properties of this solvent. However, the solubility in CDCl_3_ turned out to be very low for the studied compounds, so that only ^1^H NMR spectra could be obtained from very dilute solutions in this solvent. For all compounds the marked line broadening did not permit the determination of ^13^C,^1^H spin coupling constants of the pyrazole C-atoms which, in certain cases, may be employed for a rough estimation of the tautomeric composition [28–29].

The ^1^H and ^13^C NMR spectra of aminonitrile **5a** showed one set of broad signals. The ^13^C chemical shifts of the pyrazole C-atoms (δ pyrazole C3(5) 154.1 ppm, δ pyrazole C-4 73.5 ppm, and δ of the quaternary pyrazole C5(3) 140.0 ppm) resemble those of ‘fixed’ *N*-methyl compound 5-amino-1-methyl-1*H*-pyrazole-4-carbonitrile (δ C3 139.8 ppm, δ C4 72.1 ppm, δ C5 151.4 ppm). Thus, it can be assumed that isomer **5aA2** provides a marked contribution to the tautomeric mixture. However, NOE measurement between the pyrazole-CH and pyrazole NH hints at the simultaneous presence of species **5aA1**, where the protons involved are spatially close. The noticeable contribution of **5al12** is improbable due to the presence of an NH_2_ group in the ^1^H NMR spectrum. No isomers with sp^3^ hybridized ring C-atoms or excocyclic double bonds were detected at all and thus can, at the most, play a marginal role.

Methyl 3-hydroxypyrazole-4-carboxylate (**5b**) shows a distinct dynamic behavior in DMSO-*d*_6_ solution, resulting in broad lines in the ^1^H and in the ^13^C NMR spectra. Comparison of the ^13^C chemical shifts with those of the fixed *N*-methyl derivatives methyl 3-hydroxy-1-methyl-1*H*-pyrazole-4-carboxylate and methyl 5-hydroxy-1-methyl-1*H*-pyrazole-4-carboxylate exhibits a good agreement with the former. Therefore, species **5bE1** can be assumed to have the largest contribution to the overall tautomeric mixture. It is known from the literature that - given such a type of compounds – oxo-forms and forms with exocyclic double bonds do not play an important role. The corresponding ethyl ester **5c** exhibits the same characteristics as its methyl congener **5b**. As found with **5c** dominance of tautomer **5cA1** can also be deduced from an inspection of the ^13^C NMR spectrum in DMSO-*d*_6_ solution. In Supporting Information File 1, Tables S6 and S7 signals for compounds **5a–d** in CDCl_3_ and DMSO-*d*_6_ solution are reported whenever measurement was possible.

**Figure 6:**
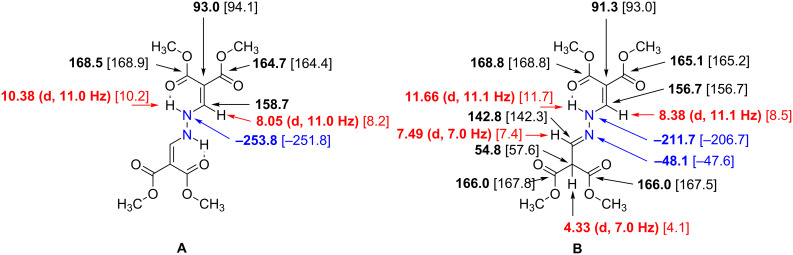
Key ^1^H (red), ^13^C (black) and ^15^N (blue) NMR chemical shifts (δ, ppm) in isomers **A** and **B** of compound **6a**. [DFT data are in square brackets.]

An interesting feature was observed in the NMR spectra of compound **6a** (Figure 6) (IUPAC name: tetramethyl 2,2'-(1,2-hydrazinediyldimethylylidene)dimalonate). In CDCl_3_ solution a mixture of the symmetrical form **A** and an unsymmetrical, tautomeric form **B** was observed (ratio A:B ~ 2:3, Figure 6). The symmetric structure of **A** can be derived from the fact that in the ^1^H,^15^N HMBC spectrum of **A** there is only one ^15^N resonance. This resonance consists of residual signals from the incompletely suppressed direct coupling (^1^*J*(^15^N,^1^H) ~105.9 Hz) and an additional correlation caused by the geminal ^2^*J*(**N**–N–**H**) coupling. The compound is stabilized by strong intramolecular hydrogen bonds between the NH and the ‘*cis*’-located ester carbonyl O-atom. This situation gives rise to a sharp, deshielded resonance of NH (δ 10.38 ppm), which is split into a doublet (^3^*J* = 11.0 Hz) due to the coupling with the alkene-H (δ 8.05 ppm). The ester moiety involved in the intramolecular hydrogen bond exhibits a marked downfield shift for C=O (δ 168.5 ppm) in comparison to the other ester carbonyl C-atom (δ 164.7 ppm), which is typical for such a situation [30]. The small chemical shift for the quaternary alkene C-atom (δ 93.0 ppm) is characteristic for a polarized C=C bond in this push–pull configuration. One half of the isomer **B** shows the same characteristics as **A** , that is, the intramolecular hydrogen bond between NH and the ester C=O. However, the second half of the molecule has two equivalent ester moieties and a C=N bond instead of a C=C bond leading to an sp^3^ hybridized C-atom (δ 54.8 ppm) between the equivalent methyl ester groups. The two different N-atoms in **B** have ^15^N chemical shifts of −48.1 ppm (C=N) and −211.7 ppm (=N–NH) (Figure 6).

### Quantum-chemical calculations

To support the experimental results were also performed DFT (Density Functional Theory method) calculations by using the B3LYP hybrid functional [31–33] and 6-311++G** basis sets of the Gaussian basis set library [34]. The SCRF theory via the IEFPCM (Integral Equation Formalism Polarizable Continuum Model) model including the effect of the solution was applied to optimize the geometries in (m)ethanol and DMSO. The stability of the obtained structures was proven by vibrational analysis (no imaginary vibrations).

NMR chemical shifts δ (in ppm) were evaluated from the corresponding absolute shieldings σ calculated for vacuum geometry by using the Gauge-Independent Atomic Orbital (GIAO) method [35] at B3LYP/6-311++G** level of theory according to the linear equation

[1]
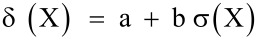


with *a* = 31.0 ± 0.8 ppm , *b* = −0.97 ± 0.03 for X = ^1^H [36], *a* = 175.7 ± 0.2 ppm , *b* = −0.963 ± 0.003 for X = ^13^C [37] and *a* = −152.0 ± 1.1 ppm , *b* = −0.946 ± 0.008 for X = ^15^N [37]. The Gaussian09 [34] software was used for all calculations.

Products **5** are formed during reactions in which the proportions of tautomers can theoretically correspond to the proportion of tautomers by the Boltzmann distribution obtained from theoretical calculations at 293.15 K. However, the calculated proportion of tautomers does not include the effects of the reaction conditions and kinetics factors on the final proportion of individual tautomers in solution.

Total electronic IEFPCM-B3LYP/6-311++G** energies with zero-point corrections *E*(DFT) and relative energies of tautomers Δ*E*(DFT) of studied compounds are summarized in Supporting Information File 1, Tables S8–S11. The most stable structural form in the **5a** series is the **5aA1** tautomer (56% in ethanol) closely followed by the **5aA2** tautomer (44% in ethanol), which is less stable in both ethanol and DMSO by only 0.6 and 0.4 kJ·mol^−1^, respectively. The remaining tautomers are probably absent in solution because their energies are more than 85 kJ·mol^−1^ higher than the **5aA1** tautomer in both solutions (Supporting Information File 1, Table S8). This is in agreement with experimental NMR spectra.

For the **5b** series the most stable tautomer is **5bE1** (83% in methanol) followed by **5bE2** (17% in methanol), which is less stable by approximately 4 kJ·mol^−1^ both in methanol and DMSO. The remaining tautomers are again probably absent in both solutions because their energies are significantly higher in comparison with the most stable **5bE1** tautomer. The tautomers without hydrogens at pyrazole nitrogen atoms (**5bE3**, **5bE4**, **5bE5**, **5bO45**, **5bE4Z** and **5bE4E**) exhibit the lowest stability (energy difference is ca 60 kJ mol^−1^) (Supporting Information File 1, Table S9). This is in agreement with experimental NMR spectra.

As expected, in the case of the **5c** series the most stable tautomer is **5cE1** (83% in ethanol) followed by **5cE2** (17% in ethanol), which is less stable by approximately 4 kJ·mol^−1^ both in ethanol and DMSO, similar to the **5b** series. The remaining tautomers are not present in solution and the stability dependence on the presence of hydrogen at pyrazole nitrogen atoms is similar as above (Supporting Information File 1, Table S10). This is in agreement with experimental NMR spectra.

In the **5d** series, the most stable tautomer is **5dA2** (70% in ethanol) followed by **5dA1** (30%) which is less stable by 2 kJ·mol^−1^ both in ethanol and DMSO. The remaining tautomers have no hydrogens at pyrazole nitrogen atoms and their energies are higher by more than 95 kJ·mol^−1^ higher compared to the two most stable tautomers (Supporting Information File 1, Table S11).

All stable tautomers of the **5a–d** series (Supporting Information File 1, Tables S12 and S13) present in solutions have one hydrogen atom at one of the pyrazole nitrogen atoms and, as indicated by the calculated NMR spectra in DMSO, the shift of this hydrogen should be 7.9–8.7 ppm. The single hydrogen at the pyrazole carbons (the same carbon atom in all tautomers of all series) has a shift of 7.2–7.7 ppm and its carbon has a shift of 126.4–143.3 ppm. The calculated shift differences are insufficient to identify the tautomer in solutions and can only be used to exclude some tautomers.

**Figure 7:**
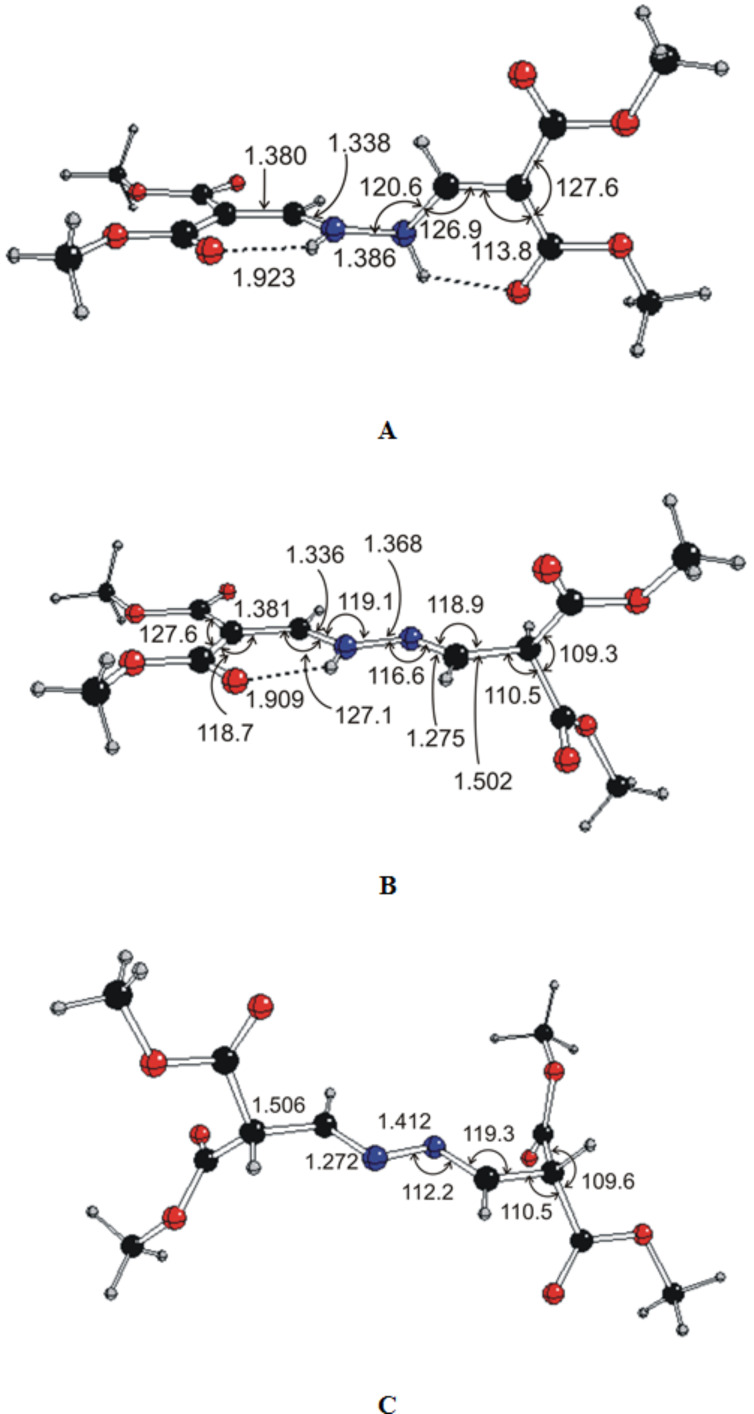
DFT optimized isomers of compound **6a**. (Distances are in Å, angles are in degrees.)

Measured NMR spectra indicate two tautomers of compound **6a** in chloroform solutions which differ in a single H atom position within the central >C–C–N–N–C–C< chain (at N or at tertiary C atom). A third tautomer with both H atoms at tertiary C atoms was not detected. DFT geometry optimization confirmed the existence of all three tautomers (Figure 7, Supporting Information File 1, Tables S2–S5 and S14) but because of energy reasons the relative population of the C conformer must be vanishing in agreement with experimental data. This was confirmed by calculated NMR spectra (Supporting Information File 1, Table S15). Nevertheless, DFT data indicate a much lower proportion of the B conformer than implied by the experimental data. In contrast to the A tautomer, the B and C tautomers have planar >C–C–N–N–C–C< chains. X-ray and DFT optimized structures of the A tautomer exhibit significant differences in torsional angles only (see Supporting Information File 1, Tables S2–S5) ascribable to solid state effects. The accuracy of the calculated NMR data is insufficient to distinguish between possible conformers of individual tautomers and so we did not performed a full conformational analysis.

## Conclusion

Activated enol ethers **3** derived from trialkyl orthoformate and active methylidene compounds such as diesters, dinitriles of malonic acid or pentane-2,4-dione are excellent three-carbon synthons. They act as trifunctional electrophiles for the syntheses of tautomeric pyrazoles when reacted with hydrazine thereby obtaining 3,4-disubstituted pyrazoles, namely 3-amino-1*H*-pyrazole-4-carbonitrile (**5a**), alkyl 3-hydroxypyrazole-4-carboxylates **5b, 5c** or 1-(3-methyl-1*H*-pyrazol-4-yl)ethanone (**5d**). The reversed addition of the enol ethers to hydrazine hydrate the formation of bis-enehydrazine **6** product was observed in two tautomeric forms: with symmetrical and unsymmetrical structures of the ethylene substituent. This structure has been established by X-ray analysis. The tautomeric behavior of these materials has been modelled by using DFT quantum-chemical calculations. The most stable structural forms are **5aA1, 5aA2**, **5bE1, 5bE2**, **5cE1**, **5cE2**, **5dA1** and **5dA2**.

As expected, NH-pyrazoles of type **5** exhibit a pronounced dynamic behavior in solution due to prototropic tautomerism. This results in the broadening of marked lines thereby preventing the identification of individual species. However, a comparison of the chemical shifts in **5** and the corresponding ‘fixed’ *N*-methyl derivatives reveals the contribution of distinct, individual tautomers to the overall situation. The structure of bis-enehydrazine **6a** – unambiguously determined by X-ray analysis – is in accord with the NMR data. Moreover, the presence of a second, asymmetric form of **6a** in CDCl_3_ solution was established by NMR. A tautomer C with an azine (C=N–N=C) structure has not been observed.

## Supporting Information

File 1 Experimental section and Tables S1–S15.

## References

[1] Milata V, Rádl S, Voltrová S (2008). Science of Synthesis Product Subclass: Enol Ethers (acyclic, cyclic, i.e. endocyclic C=C-O- unit.

[2] Milata V (2001). Aldrichimica Acta.

[3] Saloň J, Milata V, Gatial A, Prónayová N, Leško J, Černuchová P, Vo-Thanh G, Loupy A (2005). Eur J Org Chem.

[4] Kornis G I (2001). Kirk-Othmer Encyklopedia of Chemical Technology: Pyrazoles, pyrazolines, and pyrazolones.

[5] Elguero J, Marzin C, Katritzky A R (1976). The Tautomerism of Heterocycles.

[6] Minkin V I, Garnovskii A D, Elguero J, Katritzky A R, Denisko O V (2000). Adv Heterocycl Chem.

[7] Černuchová P, Vo-Thanh G, Milata V, Loupy A, Jantová S, Theiszová M (2005). Tetrahedron.

[8] Xu J, Liu H, Li G, He Y, Ding R, Wang X, Feng M, Zhang S, Chen Y, Li S (2011). Bioorg Med Chem Lett.

[9] Reddy G J, Sailaja S, Manjula D, Rao K S, Khalilullah M, Latha D (2005). Heterocycl Commun.

[10] Shaikh A C, Chen C (2008). J Labelled Compd Radiopharm.

[11] Nagahara K, Takagi K, Ueda T (1976). Chem Pharm Bull.

[12] Takagi K, Nagahara K, Ueda T (1970). Chem Pharm Bull.

[13] Kim D C, Lee Y R, Yang B-S, Shin K J, Kim D J, Chung B Y, Yoo K H (2003). Eur J Med Chem.

[14] Guillou S, Janin Y L (2010). Chem–Eur J.

[15] Ishimaru T (1957). Yakugaku Zasshi.

[16] Ruhemann S (1894). Ber Dtsch Chem Ges.

[17] Ruhemann S (1896). Ber Dtsch Chem Ges.

[18] Al-Jallo H N, Al-Khasab A, Sallomi I G (1972). J Chem Soc, Perkin Trans 1.

[19] Kitade Y, Hirota K, Maki Y (1993). J Chem Res, Miniprint.

[20] Mitkidou S, Stephanidou-Stephanatou J, Stephopoulou H (1993). J Heterocycl Chem.

[21] Menichi G, Boutar M, Kokel B, Takagi K, Hubert-Habart M (1986). J Heterocycl Chem.

[22] Nagarajan K, Arya V P, Shenoy S J (1986). J Chem Res, Miniprint.

[23] Panizzi C, Benati O (1946). Gazz Chim Ital.

[24] Elnagdi M H, Sadek K U, Galil F M A, Hassan S M E (1988). Arch Pharm.

[25] Nagahara K, Kawano H, Sasaoka S, Ukawa C, Hirama T, Takada A, Cottam H B, Robins R K (1994). J Heterocycl Chem.

[26] (2009). CrysAlisPro.

[27] Sheldrick G M (2008). Acta Crystallogr, Sect A.

[28] Holzer W, Schmid E (1995). J Heterocycl Chem.

[29] Holzer W, Krca I (2003). Heterocycles.

[30] Wang J-H, Shen Y-Q, Yu C-X, Si J-H (2000). J Chem Soc, Perkin Trans 1.

[31] Becke A D (1988). Phys Rev A.

[32] Lee C, Yang W, Parr R G (1988). Phys Rev B.

[33] Becke A D (1993). J Chem Phys.

[34] (2009). Gaussian 09.

[35] Wolinski K, Hinton J F, Pulay P (1990). J Am Chem Soc.

[36] Silva A M S, Sousa R M S, Jimeno M L, Blanco F, Alkorta I, Elguero J (2008). Magn Reson Chem.

[37] Blanco F, Alkorta I, Elguero J (2007). Magn Reson Chem.

