# Supplementary material for: Use of activated enol ethers in the synthesis of pyrazoles: reactions with hydrazine and a study of pyrazole tautomerism
Source: Beilstein J Org Chem. 2014 Apr 1;10:752–60. doi: 10.3762/bjoc.10.70 (PMC3999851; doi:10.3762/bjoc.10.70)
Supplement: File 1 — Experimental section and Tables S1–S15. [file Beilstein_J_Org_Chem-10-752-s001.pdf]

Supporting Information

for

Use of activated enol ethers in the synthesis of pyrazoles: reactions with hydrazine and a study of pyrazole tautomerism

Denisa Tarabová[1], Stanislava Šoralová[2,3], Martin Breza[2], Marek Fronc[2], Wolfgang Holzer[4] and Viktor Milata[1]*

Address: [1]Department of Organic Chemistry, Faculty of Chemical and Food Technology, Slovak University of Technology, Radlinského 9, SK-812 37 Bratislava, Slovakia, [2]Department of Physical Chemistry, Faculty of Chemical and Food Technology, Slovak University of Technology, Radlinského 9, SK-812 37 Bratislava, Slovakia, [3]Department of Pharmaceutical Chemistry, Faculty of Pharmacy, Comenius University in Bratislava, Odbojárov 10, SK-832 32 Bratislava, Slovakia and [4]Department of Drug and Natural Product Synthesis, Vienna University, Althanstrasse 14, A-1090 Vienna, Austria

E-mail: Viktor Milata - viktor.milata@stuba.sk

*Corresponding author

Dedicated to Professor Rosa María Claramunt Vallespí on the occasion of her 65th anniversary.

Experimental section and Tables S1–S15

Table S1: Crystal data of **6a**.

formula	$C_{12}H_{18}N_2O_8$
formula weight, g mol^{-1}	318.28
calculated density, g cm^{-3}	1.384
space group	Monoclinic, P2$_1$/c
A	α= γ=90°
B	91.712(3)
a, Å	7.6210(3)
b, Å	21.7720(13)
c, Å	9.2136(4)
V, Å^3	1528.07(12)
Z	4
wavelength, Å	0.71073
temperature, K	293(2)
absorption correction	analytical
μ, mm^{-1}	0.117
crystal size, mm^{-3}	0.28 x 0.44 x 0.52

Table S2: Selected interatomic distances of **6a** (Å) obtained by X-ray and DFT methods (IEFPCM-B3LYP/6-311G** in $CHCl_3$).

		X-ray	DFT			X-ray	DFT
Bond				Bond			
Atom A	Atom B			Atom A	Atom B		
C1	C2	1.470(2)	1.482	C8	O5	1.217(2)	1.229
C1	O1	1.202(2)	1.220	C8	O6	1.336(2)	1.337
C1	O2	1.347(2)	1.341	C9	O2	1.442(3)	1.439
C2	C3	1.374(2)	1.380	C10	O3	1.216(2)	1.229
C2	C10	1.458(2)	1.468	C10	O4	1.339(2)	1.337
C3	N5	1.321(2)	1.338	C11	O4	1.442(3)	1.440
N5	N6	1.390(2)	1.386	C12	O7	1.198(2)	1.220
N6	C6	1.323(2)	1.338	C12	O8	1.331(2)	1.341
C6	C7	1.376(2)	1.379	C13	O8	1.438(3)	1.439
C7	C8	1.454(2)	1.468	C14	O6	1.438(2)	1.440
C7	C12	1.472(2)	1.482				

Table S3: Hydrogen bonds geometry of **6a** (Å, °) obtained by X-ray and DFT methods (IEFPCM-B3LYP/6-311G** in $CHCl_3$).

D-H···A	D-H		H...A		D···A		D-H···A	
	X-ray	DFT	X-ray	DFT	X-ray	DFT	X-ray	DFT
N5-H5A...O3	0.86	1.021	2.02	1.923	2.6393(18)	2.635	127.9	124.2
N5-H5A···O4[1]	0.86	-	2.58	-	3.0574(19)	-	116.1	-
N6-H6A···O5	0.86	1.020	2.07	1.931	2.6774(18)	2.639	126.8	123.8
N6-H6A···O5[2]	0.86	-	2.24	-	2.9419(19)	-	139.3	-
Symmetry code: (1) x, -y+1/2, z+1/2; (2) -x+1, -y, -z+1								

Table S4: Bond angles of **6a** (°) obtained by X-ray and DFT methods (IEFPCM-B3LYP/6-311G** in $CHCl_3$).

			X-ray	DFT				X-ray	DFT
C2	C1	O1	126.6(2)	123.3	H9A	C9	O2	109.4(2)	110.6
C2	C1	O2	111.0(1)	114.5	C2	C10	O3	123.2(1)	122.5
O1	C1	O2	122.3(2)	122.1	C2	C10	O4	115.3(1)	116.0
C1	C2	C3	117.5(1)	113.8	O3	C10	O4	121.4(1)	121.5
C1	C2	C10	123.2(1)	127.6	H11C	C11	H11B	109.4(2)	110.5
C3	C2	C10	119.1(1)	118.6	H11C	C11	H11A	109.4(2)	110.5
C2	C3	H3A	116.7(2)	118.0	H11C	C11	O4	109.5(2)	109.2
C2	C3	N5	126.4(1)	126.9	H11B	C11	H11A	109.5(2)	110.5
H3A	C3	N5	116.9(2)	115.0	H11B	C11	O4	109.5(2)	110.6
C3	N5	H5A	119.8(1)	119.5	H11A	C11	O4	109.5(2)	110.6
C3	N5	N6	120.4(1)	120.6	C7	C12	O7	124.0(2)	123.3
H5A	N5	N6	119.8(1)	119.1	C7	C12	O8	114.2(1)	114.5
N5	N6	H6A	119.9(1)	119.1	O7	C12	O8	121.7(2)	122.1
N5	N6	C6	120.2(1)	120.3	H13C	C13	H13B	109.5(2)	110.5
H6A	N6	C6	119.9(2)	119.6	H13C	C13	H13A	109.5(2)	110.5
N6	C6	H6A	116.5(2)	114.9	H13C	C13	O8	109.5(2)	109.2
N6	C6	C7	126.9(2)	127.2	H13B	C13	H13A	109.4(2)	110.5
H6A	C6	C7	116.6(2)	117.9	H13B	C13	O8	109.5(2)	110.6
C6	C7	C8	119.2(1)	118.6	H13A	C13	O8	109.5(2)	110.6
C6	C7	C12	114.3(1)	113.7	H14C	C14	H14B	109.4(3)	110.5
C8	C7	C12	126.4(1)	127.7	H14C	C14	H14A	109.5(3)	110.5
C7	C8	O5	122.9(2)	122.5	H14C	C14	O6	109.4(2)	109.2
C7	C8	O6	115.2(1)	116.0	H14B	C14	H14A	109.5(3)	110.5
O5	C8	O6	121.8(2)	121.5	H14B	C14	O6	109.5(2)	110.6
H9C	C9	H9B	109.5(2)	110.5	H14A	C14	O6	109.5(2)	110.6

H9C	C9	H9A	109.4(2)	110.5	C1	O2	C9	116.7(2)	115.8
H9C	C9	O2	109.5(2)	109.2	C10	O4	C11	115.5(1)	116.0
H9B	C9	H9A	109.5(2)	110.5	C8	O6	C14	116.4(2)	116.0
H9B	C9	O2	109.5(2)	110.6	C12	O8	C13	116.1(2)	115.8

Table S5: Torsion angles of **6a** (°) obtained by X-ray and DFT methods (IEFPCM-B3LYP/6-311G** in $CHCl_3$).

				X-ray	DFT
O1	C1	C2	C3	152.28(17)	2.3
O2	C1	C2	C3	-25.2(2)	-177.6
O1	C1	C2	C10	-23.3(3)	-177.5
O2	C1	C2	C10	159.22(16)	2.7
C10	C2	C3	N5	2.1(3)	-1.3
C1	C2	C3	N5	-173.67(15)	178.9
C2	C3	N5	N6	174.09(15)	174.0
C3	N5	N6	C6	-77.0(2)	111.9
N5	N6	C6	C7	177.71(16)	172.5
N6	C6	C7	C8	-1.8(3)	-1.9
N6	C6	C7	C12	-179.04(16)	178.6
C6	C7	C8	O5	15.9(3)	2.1
C12	C7	C8	O5	-167.20(18)	-178.4
C6	C7	C8	O6	-161.27(16)	-176.9
C12	C7	C8	O6	15.6(3)	2.5
C3	C2	C10	O3	0.6(3)	1.7
C1	C2	C10	O3	176.09(16)	-178.5
C3	C2	C10	O4	179.39(14)	-177.7
C1	C2	C10	O4	-5.1(2)	2.1
C6	C7	C12	O7	11.1(3)	2.3
C8	C7	C12	O7	-165.9(2)	-177.1
C6	C7	C12	O8	-167.42(15)	-177.3
C8	C7	C12	O8	15.5(3)	3.2
O1	C1	O2	C9	-7.8(3)	0.2
C2	C1	O2	C9	169.87(17)	-179.9
O3	C10	O4	C11	1.2(3)	0.3
C2	C10	O4	C11	-177.66(18)	179.7
O5	C8	O6	C14	-2.3(3)	0.6
C7	C8	O6	C14	174.89(19)	179.6
O7	C12	O8	C13	0.3(3)	0.2
C7	C12	O8	C13	178.90(18)	179.8

Table S6: ^{1}H NMR spectra in DMSO-d_6/$CDCl_3$ solution for compounds **5a–d**.

**5a**	**NH**	**H-5**	**NH_2**		
	12.09br s/-	7.70/7.69	6.01/4.20		
**5b**	**NH**	**OH**	**H-5**	**OCH_3**	
	9.0 br s	13.5/-	7.89/-	3.65	
**5c**	**NH**	**OH**	**H-5**	**OCH_2**	**CH_3**
	8.9	13.5	7.75/7.86	4.34 (q, J = 7.2 Hz, 2H, OCH_2)/ 4.13 (q, J = 7.1 Hz, 2H, OCH_2)	1.37 (t, J = 7.2 Hz, 3H, CH_3)/ 1.21 (t, J = 7.1 Hz, 3H, CH_3)
**5d**	**H-5**	**$COCH_3$**	**CH_3e**		
	7.95s	2.56s	2.43s		

Table S7: ^{13}C NMR spectra in DMSO-d_6/$CDCl_3$ solution for compounds **5a–d**.

**5a**	**C-3**	**C-4**	**C-5**	**CN**		
	154.1	73.5	140.0	115.4		
**5b**	**CO**	**C-4**	**C-5**	**CO_2CH_3**	**OCH_3**	
	160.3	96.7	134.5	163,1 (O-CO,^{3}J(CO,OMe) = 4.0 Hz)	50.5 (^{1}J = 146.5 Hz)	
**5c**	**CO**	**C-4**	**C-5**	**CO_2Et**	**OCH_2**	**CH_3**
	160.1	96.9	134.2	162.7	58.8	14.4
**5d**	**CO**	**C-3**	**C-4**	**C-5**	**Me**	**Me**
	193.7	145.4	120.0	139.5	28.5	12.2

Table S8: Total electronic IEFPCM-B3LYP/6-311++G** energies with zero-point corrections *E*(DFT), relative energies Δ*E* of different tautomeric forms of compounds **5a**, and percentage of its tautomers by the Boltzmann distribution at 298.15 K.

	Ethanol			***DMSO***		
**Tautomer**	***E*(DFT) (Hartree)**	***ΔE*(DFT) ($kJ\cdot mol^{-1}$)**	**Percentual proportion (%)**	***E*(DFT) (Hartree)**	***ΔE*(DFT) ($kJ\cdot mol^{-1}$)**	**Percentual proportion (%)**
**5aA1**	-373.84320	0.00	56	-373.84370	0.00	54
**5aA2**	-373.84297	0.62	44	-373.84356	0.37	46
**5aA3**	-373.77599	176.45	0	-373.77646	176.53	0
**5aA4**	-373.80454	101.48	0	-373.80518	101.12	0
**5aA5**	-373.80608	97.44	0	-373.80657	97.48	0
**5aI12**	-373.81010	86.89	0	-373.81100	85.85	0
**5aI24**	-373.80122	110.21	0	-373.80170	110.26	0
**5aI45**	-373.78504	152.68	0	-373.78555	152.64	0

Table S9: Total electronic IEFPCM-B3LYP/6-311++G** energies with zero-point corrections *E*(DFT), relative energies Δ*E* of different tautomeric forms of compounds **5b** (R = Me), and percentage of its tautomers by the Boltzmann distribution at 298.15 K.

	Methanol			***DMSO***		
**Tautomer**	***E*(DFT) (Hartree)**	***ΔE*(DFT) ($kJ\cdot mol^{-1}$)**	**Percentual proportion (%)**	***E*(DFT) (Hartree)**	***ΔE*(DFT) ($kJ\cdot mol^{-1}$)**	**Percentual proportion (%)**
**5bE1**	-529.36459	0.00	83	-529.36482	0.00	84
**5bE2**	-529.36308	3.95	17	-529.36324	4.14	16
**5bE3**	-529.29634	179.14	0	-529.29658	179.13	0
**5bE4**	-529.32136	113.47	0	-529.32168	113.25	0
**5bE5**	-529.32168	112.63	0	-529.32187	112.73	0
**5bO12**	-529.35870	15.44	0	-529.35917	14.82	0
**5bO24**	-529.33924	66.54	0	-529.33953	66.38	0
**5bO45**	-529.32182	112.26	0	-529.32206	112.25	0
**5bE4Z**	-529.31830	121.50	0	-529.31864	121.22	0
**5bE4E**	-529.31224	137.41	0	-529.31274	136.71	0
**5bO24Z**	-529.34696	46.27	0	-529.34718	46.30	0
**5bO24E**	-529.33243	84.42	0	-529.33268	84.36	0

Table S10: Total electronic IEFPCM-B3LYP/6-311++G** energies with zero-point corrections *E*(DFT), relative energies Δ*E* of different tautomeric forms of compounds **5c** (R = Et), and percentage of its tautomers by the Boltzmann distribution at 298.15 K.

	Ethanol			***DMSO***		
**Tautomer**	***E*(DFT) (Hartree)**	***ΔE*(DFT) (kJ· mol^{-1})**	**Percentual proportion (%)**	***E*(DFT) (Hartree)**	***ΔE*(DFT) (kJ·mol^{-1})**	**Percentual proportion (%)**
**5cE1**	-568.66607	0.00	83	-568.66655	0.00	85
**5cE2**	-568.66456	3.96	17	-568.66489	4.36	15
**5cE3**	-568.59775	179.34	0	-568.59823	179.35	0
**5cE4**	-568.62188	115.98	0	-568.62250	115.63	0
**5cE5**	-568.62200	115.68	0	-568.62238	115.95	0
**5cO12**	-568.65942	17.46	0	-568.66039	16.17	0
**5cO24**	-568.64856	45.95	0	-568.64905	45.94	0
**5cO45**	-568.62210	115.41	0	-568.62258	115.44	0
**5cE4Z**	-568.61893	123.72	0	-568.61961	123.22	0
**5cE4E**	-568.61243	140.80	0	-568.61348	139.31	0
**5cO24Z**	-568.65208	36.71	0	-568.65245	37.02	0
**5cO24E**	-568.63455	82.74	0	-568.63505	82.69	0

Table S11: Total electronic IEFPCM-B3LYP/6-311++G** energies with zero-point corrections *E*(DFT), relative energies Δ*E* of different tautomeric forms of compounds **5d**, and percentage of its tautomers by the Boltzmann distribution at 298.15 K.

	Ethanol			***DMSO***		
**Tautomer**	***E*(DFT) (Hartree)**	***ΔE*(DFT) (kJ·mol^{-1})**	**Percentual proportion (%)**	***E*(DFT) (Hartree)**	***ΔE*(DFT) (kJ·mol^{-1})**	**Percentual proportion (%)**
**5dA1**	-418.16778	2.12	30	-418.16823	2.02	31
**5dA2**	-418.16859	0.00	70	-418.16900	0.00	69
**5dA3**	-418.11570	138.83	0	-418.11609	138.91	0
**5dA4**	-418.12483	114.86	0	-418.12545	114.34	0
**5dA5**	-418.12169	123.11	0	-418.12194	123.53	0
**5dH4Z**	-418.12902	103.86	0	-418.12984	102.82	0
**5dH4E**	-418.13180	96.57	0	-418.13253	95.74	0

Table S12: DFT calculated ^{1}H NMR spectra in DMSO for compounds **5a–d**.

**5aA1**	**$H_{N\text{-arom}}$**	**$H_{C\text{-arom}}$**	**NH_2**		
	8.0	7.2	3.5 / 3.8		
**5aA2**	**$H_{N\text{-arom}}$**	**$H_{C\text{-arom}}$**	**NH_2**		
	7.9	7.3	3.4 / 4.0		
**5bE1**	**$H_{N\text{-arom}}$**	**$H_{C\text{-arom}}$**	**H_O**	**OCH_3**	
	8.1	7.2	8.2	3.5 / 3.8	
**5bE2**	**$H_{N\text{-arom}}$**	**$H_{C\text{-arom}}$**	**H_O**	**OCH_3**	
	8.3	7.4	9.1	3.6 / 3.8	
**5cE1**	**$H_{N\text{-arom}}$**	**$H_{C\text{-arom}}$**	**H_O**	**OCH_2**	**CH_3**
	8.1	7.2	8.2	4.1	1.2 / 1.4
**5cE2**	**$H_{N\text{-arom}}$**	**$H_{C\text{-arom}}$**	**H_O**	**OCH_2**	**CH_3**
	8.3	7.5	9.1	4.1	1.1 / 1.5
**5dA1**	**$H_{N\text{-arom}}$**	**$H_{C\text{-arom}}$**	**$COCH_3$**		**CH_3**
	8.6	7.5	2.0 / 2.4		2.4 / 2.6
**5dA2**	**$H_{N\text{-arom}}$**	**$H_{C\text{-arom}}$**	**$COCH_3$**		**CH_3**
	8.7	7.7	2.0 / 2.5		1.8 / 2.7

Table S13: DFT calculated ^{13}C NMR spectra in DMSO for compounds **5a–d**.

**5aA1**	**$_{H}C_{arom}$**	**C_{CN}**	**C_{NH2}**	**$C_{\equiv N}$**		
	131.6	81.7	157.6	111.6		
**5aA2**	**$_{H}C_{arom}$**	**C_{CN}**	**C_{NH2}**	**$C_{\equiv N}$**		
	143.3	77.3	149.1	111.7		
**5bE1**	**$_{H}C_{arom}$**	**C_{COOCH3}**	**C_{OH}**	**$C_{=O}$**	**C_{H3}**	
	126.6	98.1	165.4	166.8	51.4	
**5bE2**	**$_{H}C_{arom}$**	**C_{COOCH3}**	**C_{OH}**	**$C_{=O}$**	**C_{H3}**	
	138.4	94.6	159.1	166.8	51.3	
**5cE1**	**$_{H}C_{arom}$**	**C_{COOEt}**	**C_{OH}**	**$C_{=O}$**	**C_{H2}**	**C_{H3}**
	126.4	98.4	165.5	166.3	62.8	13.6
**5cE2**	**$_{H}C_{arom}$**	**C_{COOEt}**	**C_{OH}**	**$C_{=O}$**	**C_{H2}**	**C_{H3}**
	138.3	94.7	159.1	166.4	62.8	13.6
**5dA1**	**$_{H}C_{arom}$**	**C_{COCH3}**	**C_{CH3}**	**$C_{=O}$**	**C_{H3CO}**	**C_{H3}**
	129.2	121.0	153.8	190.0	26.5	15.4
**5dA2**	**$_{H}C_{arom}$**	**C_{COCH3}**	**C_{CH3}**	**$C_{=O}$**	**C_{H3CO}**	**C_{H3}**
	142.1	120.3	143.5	191.2	27.1	13.1

Table S14: Total electronic IEFPCM-B3LYP/6-311++G** energies with zero-point corrections *E*(DFT), relative energies Δ*E* of different tautomeric forms of tetramethyl 2,2'-(1,2-hydrazinediyldimethylylidene)dimalonate **6a**, and percentage of its tautomers by the Boltzmann distribution at 298.15 K.

Tautomer	*E*(DFT) (Hartree)	*ΔE*(DFT) ($kJ\cdot mol^{-1}$)	Percentual proportion (%)
**6aA**	-1178.27183	0.00	97
**6aB**	-1178.26846	8.84	3
**6aC**	-1178.25038	56.30	0

Table S15: Experimental and DFT calculated NMR spectra for various tautomers of compound **6a** in $CDCl_3$ solutions (for atom numbering see Figure 5).

	Tautomer 6aA		Tautomer 6aB		Tautomer 6aC
Atom	Exp.	DFT	Exp.	DFT	DFT
C1	164.7	164.4	165.1	165.2	167.5
C2	93.0	94.1	91.3	93.0	58.5
C3	158.7	160.4	156.7	156.7	163.4
C6	158.7	160.4	142.8	142.3	164.5
C7	93.0	94.4	54.8	57.6	56.9
C8	168.5	168.9	166.0	167.8	166.4
C10	168.5	168.7	168.8	168.8	167.0
C12	164.7	164.6	166.0	167.5	167.2
N5	-253.8	-251.7	-211.7	-206.7	-8.0
N6	-253.8	-251.8	-48.1	-47.6	-12.0
H2A	-	-	-	-	4.2
H3A	8.05	8.2	8.38	8.5	8.2
H5A(N)	10.38	10.2	11.66	11.7	-
H6A(N)	10.38	10.2	-	-	-
H6A(C)	8.05	8.2	7.49	7.4	8.3
H7A	-	-	4.33	4.1	4.3

Experimental section

Experimental

Melting points were determined by using a Kofler hot plate and are uncorrected. Solvents were freshly distilled and dried before use. Three of the four enol ethers used (MMMM = dimethyl methoxymethylidenemalonate, EMME = diethyl ethoxymethylidenemalonate, and EMMN = ethoxymethylidenemalononitrile) in the reaction were commercially available. The fourth enol ether EMAA = 3-ethoxymethylidenepentan-2,4-dione was prepared according to a known procedure [1]. IR spectra were recorded on a FTIR Nicolet NEXUS 470 spectrophotometer in solid phase by using ATR) over the region 4500–600 cm^{-1}. ^{1}H and ^{13}C NMR spectra were measured in DMSO-d_6 and $CDCl_3$ solution by using a Varian INOVA 600 spectrometer (for ^{1}H NMR 599.782 MHz and for ^{13}C NMR 150.830 MHz) at 25 °C, a Varian UnityPlus 300 spectrometer (299.95 MHz for ^{1}H, 75.43 MHz for ^{13}C), a Bruker Avance III 400 spectrometer (400.23 MHz for ^{1}H, 100.65 MHz for ^{13}C), and a Bruker Avance 500 spectrometer (500.13 MHz for ^{1}H, 125.77 MHz for ^{13}C) at 25 °C. The center of the solvent signal was used as an internal standard, which was related to TMS with δ 7.26 ppm (^{1}H, $CDCl_3$), δ 2.49 ppm (^{1}H, DMSO-d_6), δ 77.0 ppm (^{13}C, $CDCl_3$), and δ 39.5 ppm (^{13}C, DMSO-d_6). ^{15}N NMR spectra (50.68 MHz or 40.56 MHz) were obtained on a Bruker Avance 500 or a Bruker Avance III 400 spectrometer with a 'directly' detecting broadband observe probe (BBFO) and were referenced against external nitromethane. The digital resolution was 0.25 Hz/data point in the ^{1}H spectra and 0.4 Hz/data point in the ^{13}C NMR spectra. Assignment of signals was accomplished by using gs-HSQC or gs-HMBC spectroscopic analysis or considering ^{1}H-coupled ^{13}C NMR spectra (gated decoupling) [2]. Chemical shifts (δ-scale) are quoted in parts per million and the following abbreviations are used: s = singlet; d = doublet; t = triplet; q = quartet; br = broad.

General procedure for preparation of 4b, 4c

To a stirred (m)ethanolic solution of hydrazine hydrate (6.4 mmol) was added (m)ethoxymethylidenemalonate **3b,3c** (6,4 mmol) dropwise at rt. The reaction mixture was stirred at this temperature for 10 min. After (m)ethanol evaporation, the solid residue was purified by column chromatography.

Dimethyl 2-(hydrazinylmethylidene)malonate (4b)

Colorless crystalline product, yield 95%, mp 136-138 °C; IR (ν_{max}, cm^{-1}): 791, 1083, 1253, 1596, 1655, 3263. UV VIS (DMSO, nm) λ_{max} 282. ^{1}H NMR (300 MHz, $CDCl_3$) δ_H 3.71 (3H, s, OMe), 3.78 (3H, s, OMe), 8.05 (-CH=, d, ^{3}J = 11 Hz, 1H), 10.39 (-NH-CH, d, ^{3}J = 11 Hz, 1H). ^{13}C NMR (75 MHz, $CDCl_3$): δ_C (ppm) 51.8 (OMe), 52.1 (OMe), 92.9 (C-4), 158.9 (C-3), 165.0 (CO), 168.8 (CO). Anal. Calcd. for $C_6H_{10}N_2O_4$ (174.15): C, 41.38; H, 5.79; N, 16.09%. Found: C, 41.35; H, 5.71; N, 15.90%.

Diethyl 2-(hydrazinylmethylidene)malonate (4c)

Pale yellow solid, yield 78 %, mp 107-109 °C; IR (ν_{max}, cm^{-1}): 789, 1069, 1229, 1608, 1655, 2980, 3291, 3341. UV VIS (DMSO, nm) λ_{max} 283. ^{1}H NMR (300 MHz, $CDCl_3$): δ_H 1.37-1.29 (6H, m, CH_3), 4.30-4.15 (4H, m, OCH_2), 8.23 (d, 1H, ^3J = 11.5 Hz, -CH=C), 9.90 (d, 1H, ^3J = 11.2 Hz, –NH-CH). ^{13}C NMR (75 MHz, $CDCl_3$): δ_C (ppm) 14.3 (CH_3), 14.4 (CH_3), 59.8 (OCH_2), 60.1 (OCH_2), 88.5 (C-4), 162.1 (C-3), 165.5 (CO), 169.3 (CO). Anal. Calcd. for $C_8H_{14}N_2O_4$ (202.21): C, 47.52; H, 6.98; N, 13.85%. Found: C, 47.69; H, 6.85; N, 13.61%.

General procedure for preparation of 5a–d

To a stirred (m)ethanolic solution (only **5b** in methanol) of hydrazine hydrate (17.2 mmol) was added enol ether **3a–d** (17.2 mmol) dropwise at rt. The reaction mixture was heated under reflux for 1–1.5 h. After cooling the crude product was filtered and recrystallized from the appropriate solvent or purified by column chromatography.

3-Amino-1*H*-pyrazole-4-carbonitrile (5a)

Pale pink powder, yield 91 %, mp 168-170 °C (173-174 °C [3]). IR (ν, cm^{-1}): 716, 1034, 2238, 3239, 3340, 3414. UV VIS (DMSO, nm) λ_{max} < 250, 350. ^{1}H NMR (400 MHz, $CDCl_3$): δ_H 7.69 (1H, s, H-5), 4.20 (2H, br s, NH_2), pyrazole NH not found; ^{1}H NMR (400 MHz, DMSO-d_6): δ_H 12.09 (1H, br s, pyrazole NH), 7.70 (1H, s, H-5), 6.01 (2H, s, NH_2). ^{13}C NMR (100 MHz, DMSO-d_6): δ_C 73.5 (C-4), 115.4 (CN), 140.0 (C-5), 154.1 (C-3).). Anal. Calcd. for $C_4H_4N_4$ (108.10): C, 44.44; H, 3.73; N, 51.83%. Found: C, 44.37; H, 3.77; N, 51.66%.

Methyl 3-hydroxy-1*H*-pyrazole-4-carboxylate (5b)

White powder, yield 72 %, mp 211-213 °C (204-205 °C [4]). IR (ν, cm^{-1}) 1078, 1228, 1607, 1645, 1701, 2953, 3029, 3218. UV VIS (DMSO, nm) λ_{max} < 250. ^{1}H NMR (300 MHz, DMSO-d_6): δ_H 3.65 (3H, s, OCH_3), 7.89 (1H, s, H-5), 9.0 – 13.5 (2H, NH and OH, br s). ^{13}C NMR (75 MHz, DMSO-d_6): δ 50.5 (OCH_3, ^1J = 146.5 Hz), 134.5 (C-5), 96.7 (C-4), 160.0 (C-3), 163.1 (O-CO, ^3J(CO,OMe) = 4.0 Hz). Anal. Calcd. for $C_5H_6N_2O_3$ (142.11): C, 42.26; H, 4.26; N, 19.71%. Found: C, 42.23; H, 4.16; N, 19.58%.

Ethyl 3-hydroxy-1*H*-pyrazole-4-carboxylate (5c)

Pale yellow crystals, yield 80%, EtOH) mp 178-180 °C (185 °C [5]). IR (ν, cm^{-1}) 752, 780, 1093, 1105, 1210, 1698, 2626, 2980, 3136. UV VIS (DMSO, nm) λ_{max} < 250. ^{1}H NMR (500 MHz, $CDCl_3$): δ_H 1.37 (3H, t, J = 7.2 Hz, CH_3), 4.34 (2H, q, J = 7.2 Hz, OCH_2), 7.75 (1H, s, H-5), NH, OH not found; ^{1}H NMR (300 MHz, DMSO-d_6): δ_H 1.21 (3H, t, J = 7.1 Hz, CH_3), 4.13 (2H, q, J = 7.1 Hz, OCH_2), 7.86 (1H, s, H-5), 8.9 – 13.5 (2H, NH and OH, br s). ^{13}C NMR (75 MHz, DMSO-d_6): δ_C 14.4 (CH_3), 58.8 (OCH_2), 96.9 (C-4), 134.2 (C-5), 160.1 (C-3), 162.7 (O-CO).

Anal. Calcd. for $C_6H_8N_2O_3$ (156.14): C, 46.15; H, 5.16; N, 17.94%. Found: C, 46.25; H, 5.07; N, 17.72%.

1-(3-Methyl-1*H*-pyrazol-4-yl)ethanone (5d)

Pale yellow solid, yield 88 %, mp 59-61 °C (65-68 °C [6]). IR (ν, cm^{-1}) 945, 961, 1263, 1541, 1640, 2891, 2994, 3058, 3114, 3176. UV VIS (DMSO, nm) λ_{max} < 250. ^{1}H NMR (300 MHz, $CDCl_3$): δ_H 2.43 (3H, s, Me), 2.56 (3H, s, Me), 7.95 (1H, s, H-5). ^{13}C NMR (75 MHz, $CDCl_3$): δ_C 12.2 (Me), 28.5 (Me), 120.0 (C-4), 139.5 (C-5), 145.4 (C-3), 193.7 (CO). Anal. Calcd. for $C_6H_8N_2O$ (124.14): C, 58.05; H, 6.50; N, 20.62%. Found: C, 57.99; H, 6.44; N, 20.45%.

General procedure for preparation of 6a and 6b

To a stirred (m)ethanolic solution of (m)ethoxymethylidenemalonate (6.4 mmol) was added hydrazine hydrate (6.4 mmol) dropwise at 0 °C, and the reaction mixture was stirred at this temperature for 10 min, followed by 20 min stirring at room temperature. After evaporation of the solvent, the solid product was recrystallized from the appropriate solvent and dried.

Tetramethyl 2,2'-(1,2-hydrazinediyldimethylylidene)dimalonate (6a) (Figure 5)

Colourless crystals, yield 60 % ($CHCl_3$), mp 131-2 °C. IR (ν, cm^{-1}) 750, 783, 1039, 1109, 1211, 1547, 1589, 2622, 2980, 3131.

Isomer **A** (symmetrical): ^{1}H NMR (400 MHz, $CDCl_3$): δ_H 3.73 (6H, s, OCH_3 *trans* to NH), 3.81 (6H, s, OCH_3 *cis* to NH), 8.05 (2H, d, *J* = 11.0 Hz, =CH), 10.38 (2H, d, *J* = 11.0 Hz, NH); ^{13}C NMR (100 MHz, $CDCl_3$): δ_C 51.6 (OCH_3 *trans* to NH), 51.9 (OCH_3 *cis* to NH), 93.0 (**<u>C</u>**=CH), 158.7 (=CH), 164.7 (C=O *trans* to NH), 168.5 (C=O *cis* to NH). ^{15}N NMR (40 MHz, $CDCl_3$): δ_N -253.8 (^1J = 105.9 Hz, NH).

Isomer **B** (unsymmetrical): ^{1}H NMR (400 MHz, $CDCl_3$): δ_H 3.72 (3H, s, OCH_3 *trans* to NH), 3.80 (6H, s, OCH_3), 3.81 (3H, s, OCH_3 *cis* to NH), 4.33 (1H, d, *J* = 7.0 Hz, C-H), 7.49 (1H, d, *J* = 7.0 Hz, N=CH), 8.38 (1H, d, *J* = 11.1 Hz, 1H, =CH), 11.66 (1H, d, *J* = 11.1 Hz, 1H, NH). ^{13}C NMR (100 MHz, $CDCl_3$): δ_C 51.4 (OCH_3 *trans* to NH), 51.7 (OCH_3 *cis* to NH), 53.3 (2×OCH_3), 54.8 (**<u>C</u>**H$(CO)_2$), 91.3 (**<u>C</u>**=CH), 142.8 (N=CH), 156.7 (=CH), 165.1 (C=O *trans* to NH), 166.0 (2×C=O), 168.8 (C=O *cis* to NH). ^{15}N NMR (40 MHz, $CDCl_3$): δ_N -48.1 (C=N), -211.7 (^1J = 96.3 Hz), NH). Anal. Calcd. for $C_{12}H_{16}N_2O_8$ (316.26): C, 45.57; H, 5.10; N, 8.86%. Found: C, 45.70; H, 5.10; N, 8.78%.

Tetraethyl 2,2'-(1,2-hydrazinediyldimethylylidene)dimalonate (6b)

Pale yellow crystals, yield 45 % (EtOH), mp 119-121 °C. IR (ν, cm^{-1}) 751, 783, 918, 1114, 1519, 1539, 1580, 3329, 3369. ^{1}H NMR (300 MHz, $CDCl_3$): δ_H 1.35-1.23 (12H, m, CH_3), 4.33-4.10 (8H, m, OCH_2), 8.44 (2H, d, ^2J = 11.2 Hz, -CH=C), 9.86 (1H, d, ^3J = 11.4 Hz, -CH=C), 11.62 (1H, d, ^3J = 11.2 Hz, -NH-CH). ^{13}C NMR (75 MHz, $CDCl_3$): δ_C 14.3 (CH_3), 14.4 (CH_3), 17.0 (CH_3), 25.0 (CH_3), 59.6 (OCH_2), 59.8 (OCH_2), 60.0 (OCH_2), 60.3 (OCH_2), 88.4 (CH=<u>C</u>), 89.7

(CH=C), 155.9 (CH=C), 162.0 (CO), 165.5 (CO), 169.2 (CO). Anal. Calcd. for $C_{16}H_{24}N_2O_8$ (372.37): C, 51.61; H, 6.50; N, 7.52%. Found: C, 51.46; H, 6.46; N, 7.38%.

References

1. Wang, J. H.; Shen, Y. Q.; Yu, C. X.; Si, J. H. *J. Chem. Soc., Perkin Trans. 1* **2000,** 1455. doi:10.1039/a908958f
2. Braun,S.; Kalinowski, H.-O.; Berger.S *150 and More Basic NMR Experiments,* 2nd ed.; Wiley-VCH: Weinheim, 1998.
3. Ioannidou, H. A.; Koutentis, P. A. *Tetrahedron* **2009,** *65*, 7023.. doi:10.1016/j.tet.2009.06.041
4. Kitade, Y.; Hirota, K.; Maki, Y. *J. Chem. Res., Miniprint* **1993,** 101.
5. Guillou, S.; Janin, Y. L. *Chem.–Eur. J.* **2010,** *16*, 4669. doi:10.1002/chem.200903442
6. Nagarajan, K.; Arya, V. P.; Shenoy, S. J. *J. Chem. Res., Miniprint* **1986,** 1401.
